# Real-Life Experience of the Effects of Cladribine Tablets on Lymphocyte Subsets and Serum Neurofilament Light Chain Levels in Relapsing Multiple Sclerosis Patients

**DOI:** 10.3390/brainsci12121595

**Published:** 2022-11-22

**Authors:** Damiano Paolicelli, Maddalena Ruggieri, Alessia Manni, Concetta D. Gargano, Graziana Carleo, Claudia Palazzo, Antonio Iaffaldano, Luca Bollo, Tommaso Guerra, Annalisa Saracino, Antonio Frigeri, Pietro Iaffaldano, Maria Trojano

**Affiliations:** 1 Department of Basic Medical Sciences, Neurosciences and Sense Organs, University of Bari “Aldo Moro”, 70124 Bari, Italy; 2 Department of Biomedical Sciences and Human Oncology, University of Bari “Aldo Moro”, 70124 Bari, Italy

**Keywords:** cladribine tablet, multiple sclerosis, neurofilament, real life, no evidence of disease activity, lymphocyte subset

## Abstract

Although cladribine induces sustained reductions in peripheral T and B lymphocytes, little is known about its effect on axonal damage reduction in multiple sclerosis (MS), which could be demonstrated by assessing the serum neurofilament light chain (sNfL) levels. We investigated the reduction/reconstitution of different lymphocyte subsets (LS) by verifying the correlation with no evidence of disease activity (NEDA) and the variation in sNfL levels during cladribine treatment. We analysed 33 highly active relapsing MS patients and followed them up for 12 ± 3.3 months; blood samples were collected at treatment start (W0) and after 8, 24 and 48 weeks. Seventeen patients (60.7%) showed NEDA during the first treatment. At week 8, we observed a significant decrease in B memory cells, B regulatory 1 CD19+/CD38+ and B regulatory 2 CD19+/CD25+, a significant increase in T regulatory CD4+/CD25+, a slight increase in T cytotoxic CD3+/CD8+ and a non-significant decrease in T helper CD3+/CD4+. Starting from week 24, the B subsets recovered; however, at week 48, CD19+/CD38+ and CD19+/CD25+ reached values near the baseline, while the Bmem were significantly lower. The T cell subsets remained unchanged except for CD4+/CD25+, which increased compared to W0. The LS changes were not predictive of NEDA achievement. The sNfL levels were significantly lower at week 24 (*p* = 0.046) vs. baseline. These results could demonstrate how cladribine, by inflammatory activity depletion, can also reduce axonal damage, according to the sNfL levels.

## 1. Introduction

Multiple sclerosis (MS) is a chronic inflammatory disease involving a dysregulated immune response that results in demyelination, axonal degeneration and gliosis [1]. For many years, it has been considered an autoreactive T cell pathology [2], but recent evidence indicates that the disease also involves complex interactions among several lymphocyte subsets (LS), including B cells [3]. The involvement of B cells in MS is indicated by the presence of oligoclonal immunoglobulin bands in the cerebrospinal fluid (CSF) and the efficacy of the B-cell-depleting therapy administered to treat the disease. However, attempts to identify the antigens driving an autoreactive B cell response in the central nervous system (CNS) have been inconclusive [4]; moreover, anti-CD20 (CD: cluster of differentiation) therapy is clinically effective despite not targeting antibody-secreting plasma cells or appreciably influencing antibody levels [5]. B memory cells (Bmem) are potentially important players in MS pathophysiology because they are antigen-experienced, respond rapidly to stimulation and have a long lifespan [6]. They also tend to play a pro-inflammatory role that may drive disease activity/relapse [7]. Early lesions of the blood–brain barrier (BBB) in the MS pathogenesis promote the migration of activated T cells to the CNS [8], where they create demyelination foci. Low-level T-cell migration has also been observed in healthy individuals [9], because the CNS is an immune-privileged site where cell trafficking is controlled by the BBB and blood–CSF barrier. The migration of CD4+ T cells is more frequent than that of CD8+ T cells, leading to a higher CD4+/CD8+ ratio in CSF compared with blood [10,11].

Most MS therapies focus on immune modulation/suppression and aim to limit neuronal and axonal damage by reducing the inflammation [12]. Given the wide spectrum of new pharmacological options, the choice of disease-modifying treatments (DMTs) in MS follows two general approaches: escalation with drugs of moderate efficacy or the early use of high-efficacy treatments [13]. Immune reconstitution therapy (IRT), considered a high-efficacy treatment, can induce sustained MS remission by depleting adaptive immune system components and then allowing their recovery, which involves rebuilding a less inflammatory immune cell profile [14,15]. Examples of IRT for MS include cladribine (CLAD), alemtuzumab and autologous haematopoietic stem cell transplantation.

CLAD depletes the T and B LS with very different magnitudes and kinetics. In a work by Jacobs et al. [16], regarding a population of 309 patients, the T cells are reduced early, in a dose-dependent manner, and remain suppressed at a plateau of ~50% of the baseline level; the B cells, instead, are depleted by ~90% after the first treatment cycle, repopulate within 40 days to 80% of the pre-treatment level, and are again depleted rapidly after the second cycle to ~20% of the baseline level. CLAD has been approved for relapsing MS (RMS) based on the phase III CLARITY (CLAdRIbine Tablets treating multiple sclerosis orallY) study [17] and its extension [18], which revealed its effects on the LS, including marked reductions in CD19+ B cells. Moreover, thanks to its ability to cross the BBB [19,20], CLAD may penetrate the CNS and reach concentrations of up to 25% in the CSF of patients both with and without MS [19,20,21]. CLAD detection in CSF reinforces the likelihood that it may reduce the number of lymphocytes in both the CNS and periphery [22]. Information regarding the CLAD effects on neuronal cells is scarce, though. In an Italian in vitro study [23], CLAD induced apoptosis in the lymphocytes of healthy donors and MS patients, but neuron vitality did not seem compromised either during acute or prolonged (72 h) exposure to the drug.

The literature data have confirmed the inhibitory effect of CLAD on the migration of mononuclear cells through an in vitro BBB model [24]; this focuses on a key event in the immunopathogenesis of MS, where immunocompetent cells are involved in both degenerative and immune processes.

The mechanisms by which these immune alterations are correlated to the CLAD efficacy are not clear, yet.

Neurodegeneration plays a crucial role in MS, contributing to long-term disability. Neurofilaments, which are cytoskeletal proteins, have been recently investigated as neurodegeneration markers. Neurofilaments are expressed exclusively in neuronal cells in the CNS and periphery, and they are released into the CSF and blood in response to axonal damage. Neurofilament levels reflect axonal damage in various neurological disorders [25] and could be used as a marker for monitoring MS disease activity and response to DMT [26]. However, there is a lack of data about the longitudinal evaluation of serum neurofilament chain (sNfL) levels and IRTs in real-life studies.

The primary aim of this study, which involved a real-life setting of RMS patients, is to evaluate variations in LS and sNfL levels to clarify their potential role as biomarkers for treatment response, as well as the CLAD effectiveness and safety.

## 2. Materials and Methods

Seventy-four patients with RMS, according to the revised Lublin criteria [27], were treated with CLAD at a cumulative dose of 3.5 mg/kg. Their clinical and demographic characteristics were collected using the web application of the Italian Multiple Sclerosis Registry. CLAD was administered between 2018 and 2021 at the MS centre of the University Hospital of Bari, Italy; the patients selected before May 2019 had participated in an early-access programme. The study was performed in compliance with the International Conference on Harmonisation (ICH) and Good Clinical Practice (GCP) guidelines. The protocol was approved by the local Ethics Committee of the University of Bari “Aldo Moro”, and all the aspects of the study were compliant with the local laws and regulations; moreover, all recruited patients signed full written informed consent.

The longitudinal assessment was conducted using the Expanded Disability Status Scale (EDSS) [28] and via magnetic resonance imaging (MRI) of the brain and spinal cord; the MRI scans were acquired by an Achieva 1.5 T DS MRI system (Philips Healthcare, Netherlands) equipped with an 8-channel head coil. Baseline MRI scans were performed no more than 6 months before the treatment began and, then, at 6-month intervals (±30 days); trained neuro-radiologists counted the lesions by the visual analysis of two successive MRI images. No evidence of disease activity (NEDA) was defined by the absence of gadolinium-positive lesions and/or new, or newly enlarged, T2 lesions, the absence of relapses or worsening of EDSS score during the CLAD treatment. Active surveillance for infections was conducted monthly through a telephone questionnaire.

In a cohort of 33 RMS patients, blood samples were collected at the treatment start (W0) and after 8, 24 and 48 weeks (W8, W24 and W48, respectively). To avoid any possible confounders due to the steroid treatment in the case of relapse/MRI activity, peripheral blood samples were collected at least 30 days after the last day of corticosteroid intake. Changes in LS were analysed via flow cytometry based on the following identified markers: CD19+/CD27+ for Bmem, CD19+/CD38+ for B regulatory 1 (Br1), CD19+/CD25+ for B regulatory 2 (Br2), CD4+/CD25+ for regulatory T (Treg), CD3+/CD4+ for T helper (Th) and CD3+/CD8+ for T cytotoxic (Tc). The samples were used to define the absolute numbers of white blood cells and the LS percentage. In a cohort of 18 patients, at W0 and W24, the sNfL concentration was assessed by serum sampling.

### 2.1. Analysis of Peripheral LS

Blood samples were collected at W0, W8, W24 and W48 for immunophenotyping by flow cytometry in ethylenediaminetetraacetic acid (8.55 mg/tube). Peripheral blood samples (100 µL) were incubated with specific monoclonal antibodies (Beckman Coulter, Wycombe, UK) at room temperature (RT) for 15 min in the dark; afterward, the red cells were lysed, at RT in the dark, in a VersaLyse lysing solution (A09777, Beckman Coulter, Wycombe, UK) for 15 min and then analysed. LS were identified by the recognition of surface molecules belonging to the CD family. The samples successively underwent gating analysis (total lymphocytes and CD45+ vs. side scatter) (CD45-FITC, A07782, Beckman Coulter, Wycombe, UK), and the desired lymphocyte subpopulations were gated excluding doublets. The LS identification was based on monoclonal antibodies against Bmem CD19+/CD27+, Br1 CD19+/CD38+, Br2 CD19+/CD25+, Treg CD4+/CD25+, Th CD3+/CD4+ and Tc CD3+/CD8+ (CD19-PC7, IM3628; CD27-PE, IM2578; CD38-PE, AO7779; CD25-PE, A07774; CD4-FITC, A07750; CD3-PC5, A07749; CD8-PE, A07757; Beckman Coulter, UK). The samples were tested using a CytoFLEX flow cytometer and the CytExpert software (Beckman Coulter, Wycombe, UK).

### 2.2. sNfL Level Evaluation

The sNfL levels were assessed via an ultra-sensitive single-molecule array (SiMoA) method by using a Quanterix-SR-X™ analyser (Quanterix, Billerica, MA, USA). Blood samples were collected from each patient, centrifuged and divided into aliquots, with serum immediately frozen at −80 °C. The sNfL concentrations were measured via the commercially available SiMoA^®^ NF-light™ Advantage Kit (Quanterix, Billerica, MA, USA) as follows. The samples (25 μL) were diluted 1:4 in plates by adding 75 μL of diluent, and they were tested blindly and in duplicate. Two duplicate quality control (high- and low-concentration) samples were provided on each plate for every run. The sNfL concentrations (pg/mL) were calculated based on a standard curve derived from triplicate samples of known values according to the manufacturer’s instructions. The intra-assay coefficient of variation (CV) was computed by the SRX software from technical replicate measures of samples assayed within a single run; for these samples, the CV was below 1%.

### 2.3. Statistical Analysis

The patient characteristics at the treatment baseline are reported as mean ± standard deviation (SD), median, relative frequency or absolute frequency. Continuous variables such as EDSS and sNfL levels were calculated as mean ± SD differences assessed with the Wilcoxon signed-rank test. Linear mixed models on log values were used to evaluate the LS and innate immunity cells at 4 time points; the subjects were considered as a random factor. Estimated geometric mean and relative standard error are reported. Logarithms of marginal mean were used for innate immunity cells. The changes in LS percentages and innate immunity cells were assessed with a generalised linear mixed model. Categorical variables (e.g., relapses, presence of Gd-enhanced (Gd+) lesions, increase in T2 lesion load and global MRI disease activity) are expressed as absolute and relative frequencies, and their differences were determined with the McNemar’s test. The most frequent adverse events (infections) were expressed as relative and absolute frequencies as well. All the analyses were corrected for age and gender. The calculations were conducted with IBM SPSS Statistics 20.0 (IBM, Armonk, NY, USA), and the graphs were elaborated with the same program.

## 3. Results

### 3.1. Patients

Among the 74 RMS patients enrolled, with the mean age of 35.2 ± 11.3 years, 61 (82.4%) had previously exhibited treatment failure and 13 (17.6%) were treatment-naïve. The mean follow-up was 17 ± 7.5 months. Their clinical and demographic characteristics are listed in Table 1.

### 3.2. Effectiveness and Safety of CLAD Treatment

None of the treated patients dropped out of the study.

Relapses occurred in 12 patients (16%), with a mean time to first relapse of 6.35 ± 3.72 months. The NEDA outcomes at 12, 18 and 24 months are given in Table 2.

At least one infection was reported in 17 patients (22.97%). Infections included four cases of herpes simplex, two herpes zoster episodes, and two urinary tract infections. SARS-COV2 infections, confirmed by positive molecular swabs, occurred in 12 patients (12.2%), and one of them required hospitalisation. Among the patients who contracted the SARS-COV2 infection, the shortest time to infection onset was 13 ± 0.5 months after starting the CLAD treatment.

### 3.3. Lymphocyte Subsets

**Figure 1:**
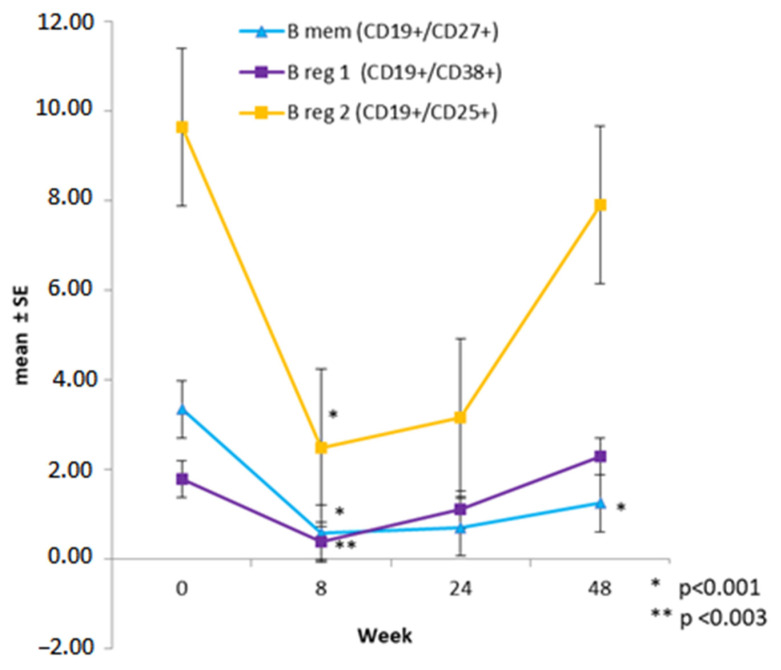
Trends in B lymphocyte subpopulations in 33 patients at specific times after starting cladribine therapy; generalised linear mixed model (* *p* < 0.001 and ** *p* < 0.003).

**Figure 2:**
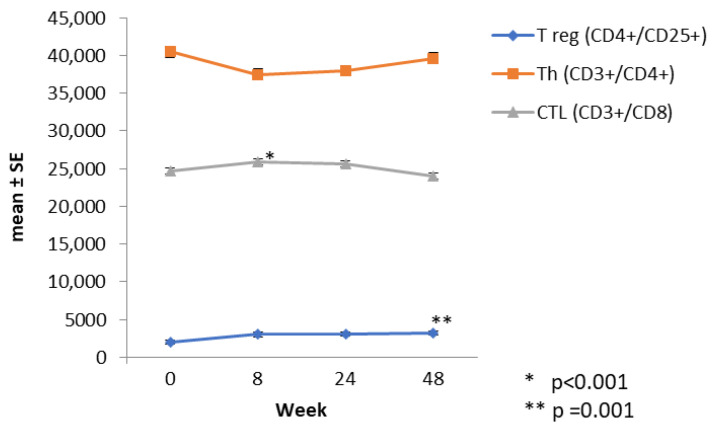
Trends in T lymphocyte subpopulations in 33 patients at specific times after starting cladribine therapy; generalised linear mixed model (* *p* < 0.001 and ** *p* = 0.001).

**Figure 3:**
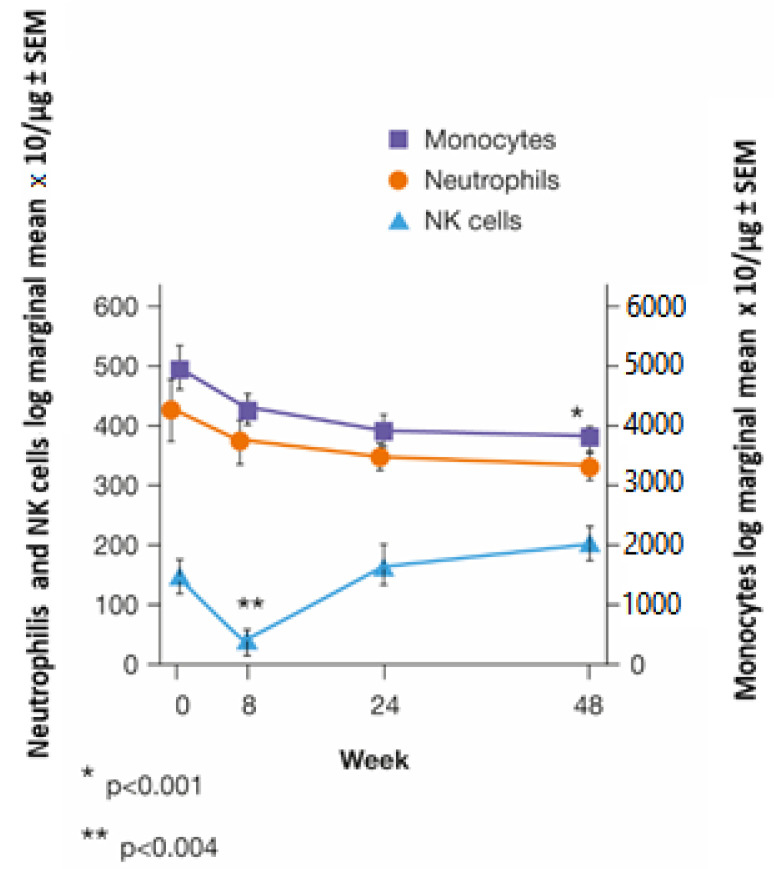
Trends in innate immune system components in 33 patients at specific times after starting cladribine therapy; generalised linear mixed model (* *p* = 0.001 and ** *p* < 0.004; NK = natural killer; SEM = standard error of the mean).

The changes in certain LS and innate immunity cells were evaluated in a subgroup of 33 patients, and the graphs in Figure 1, Figure 2 and Figure 3 were derived from the values obtained during the average follow-up period of 12 ± 3.3 months.

At W8, after receiving the first dose of CLAD, a significant decrease of 82.9% in Bmem (3.342 ± 1.11 at W0 vs. 0.571 ± 1.55 at W8; *p* < 0.001), and a decrease of 77.9% in Br1 CD19+/CD38+ (1,78 ± 1.15 at W0 vs. 0.394 ± 1.82 at W8; *p* = 0.003) and 74.36% in Br2 CD19+/CD25+ (9.638 ± 1.52 at W0 vs. 2.47 ± 1.29 at W8; *p* < 0.001) were observed. Starting from W24, the B LS exhibited a recovery trend; however, at W48, CD19+/CD38+ and CD19+/CD25+ reached values similar to the baseline, while the Bmem levels were significantly lower (1.25 ± 1.12; *p* < 0.001, with a reduction of 62.7% compared to baseline level) (Figure 1).

The T LS responded differently. There was a significant increase of 54.2% in Treg CD4+/CD25+ (1.96 ± 1.08 at W0 vs. 3.02 ± 1.11 at W8; *p* < 0.001), a slight increase of 4.95% in Tc CD3+/CD8+ (24.66 ± 1.06 at W0 vs. 25.88 ± 1.07 at W8; *p* = 0.425) and a non-significant decrease of 7.3% in Th CD3+/CD4+ (40.46 ± 1.04 at W0 vs. 37.5 ± 1.05 W8; *p* = 0.073).

At W24, the T LS remained unchanged except for CD4+/CD25+, which increased compared to W0 (3.24 ± 1.17 at W48, *p* = 0.001) (Figure 2).

The LS changes were not predictive of NEDA achievement. The Br1 CD19+/CD38+ levels were consistently higher in patients who switched to CLAD from first-line treatment vs. second-line treatment (*p* = 0.027).

### 3.4. Innate Immunity

Natural killer (NK) cells were significantly reduced at W8 (log marginal mean: 150,496 ± 30,713 at W0 vs. 45,240 ± 13,330 at W8; *p* = 0.004). Their levels returned to baseline after 24 weeks (Figure 3).

There was a gradual decrease in monocytes, which became statistically significant after 48 weeks (499,559 ± 36,908 at W0 vs. 375,858 ± 25,858 at W48; *p* = 0.001) (Figure 3).

### 3.5. sNfL Levels

**Figure 4:**
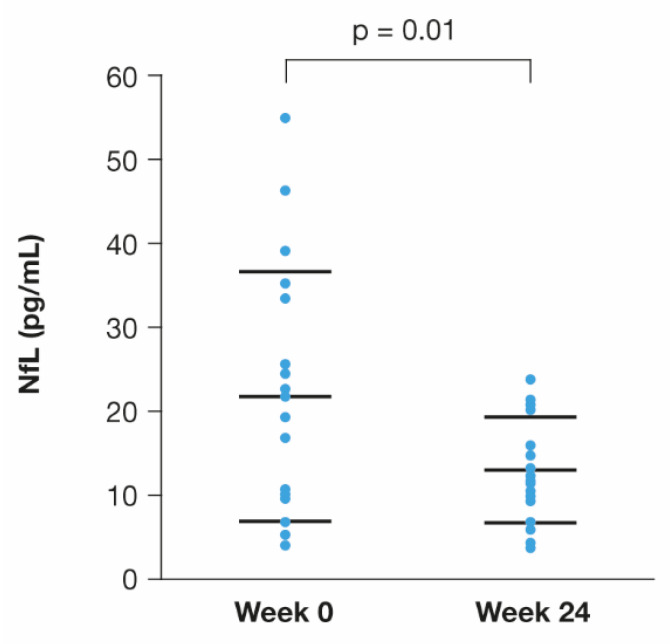
Changes from baseline in serum neurofilament light chain levels at 24 weeks after starting cladribine therapy in 18 patients with relapsing/remitting multiple sclerosis (Wilcoxon signed-rank test).

The sNfL levels significantly decreased at W24 compared with baseline (W0: 21.78 ± 14.75 pg/mL vs. W24: 13.01 ± 6.31 pg/mL; *p* = 0.01) (Figure 4).

## 4. Discussion

In recent years, several molecules capable of targeting both T and B lymphocytes have appeared in the MS landscape. CLAD provides a faster and larger reduction in B cells than that in T cells.

Here, we evaluated the temporal variation in specific LS and sNfL levels during CLAD treatment. The purpose was to detect and understand how these variables could affect the treatment effectiveness and safety in clinical practice.

In this study, Bmem underwent a statistically significant reduction starting from W8 and remained consistently below the baseline value, even a year after the beginning of the first cycle of CLAD. This reduction trend is in line with the literature [29] and is key in the disease, showing the important role of these cells in MS pathogenesis. Moreover, a study by Stuve et al. [30] reported that median CD19+ B-cell counts recovered towards baseline after week 13 but exhibited reductions of approximately 60% and 30% at week 24 and 48, respectively; thus, we wanted to analyse further subsets of B cells that were not included in the CLARITY, CLARITY extension and ORACLE-MS studies. It is interesting how molecules such as infliximab increase the Bmem levels, leading to a worsening of the disease [31]. The mechanism of action of CLAD on Bmem determines their reduction with respect to the baseline without affecting the patient antibody response. This observation has decisive implications for the therapeutic choice in RMS when patients receiving COVID-19 vaccination within 4.4 months from the last dose of CLAD develop an antibody response comparable to healthy controls and untreated MS patients [32].

Unlike Bmem, regulatory lymphocytes tended to return to baseline levels. In particular, Br1 CD19+/CD38+, after an inflection at W8, tended to rise back to baseline values at W24 and exceed them at W48; this is in line with a MAGNIFY study [33], which reported more markers and time points, with B regulatory cells having recovered by month 3 and then increased over the baseline level. In contrast, Treg CD4+/CD25+ increase significantly as early as at W8, subsequently remaining above the baseline value for all 12 months of monitoring. Treg suppress the immune self-response; in fact, their altered functioning results in autoimmune diseases such as MS [34]. B regulatory cells also apparently contribute to the complex lymphocytic network [35]. Therefore, their increase would lead to immunotolerance restoration with the consequent inhibition of self-reactive T and B lymphocytes, which both play a decisive role in the pathophysiological processes underpinning MS. Studies have further shown that the abnormal functioning of B regulatory cells can lead to autoimmunity [36]. In this cohort, the earlier repopulation of Treg compared with B regulatory lymphocytes further confirms the lower risk of occurrence of autoimmune events in CLAD-treated patients [37]. Moreover, higher Treg values defend against autoimmunity, as confirmed by several works [38,39], where the Treg levels rise after a relapse to counterbalance the inflammatory process. However, this increase, when not counterbalanced by an adequate level of Br1 CD19+/CD38+, does not prevent early relapse. Therefore, this moment of imbalance between B and T regulatory cells might be one of the time windows when patients could be more susceptible to relapse.

Regarding Tc CD3+/CD8+ and Th CD3+/CD4+ at W8, in this study, there was a slight increase in the former and a decrease in the latter, neither of which were statistically significant (*p* = 0.425 and *p* = 0.073, respectively); both values almost returned to the baseline ones at W48. This trend differs from previous work on lymphocyte variations in pooled data from the 2-year CLARITY study (and its extension) and the PREMIERE registry study [40], which showed a nadir of CD+ cells at the fourth month of treatment with a gradual recovery thereafter.

For NK cells, a statistically significant decrease was observed at W8 (W8: W0 *p* = 0.004), with recovery starting from W24 and reaching, at W48, 30% higher levels than at baseline.

The cellular expression of deoxycytidine kinase (dCK) and NT5C1 correlates with the depletion profile observed in clinical trials of CLAD therapy [41,42]. This suggests limited activity on innate immune cells and a relatively minor impact on Tc CD8+ and plasma cells, which may have implications for protection from bacterial and viral infections. The absolute number of neutrophils increased in this cohort compared with a phase III CLAD study [8]; in the reported work, as evidenced by the expression of dCK/5NT in CD56high CD62L+ and CD56low, CD62L-, NK cells should be, and indeed were, more sensitive to CLAD to a similar level to T CD4+. However, the depletion was relatively transient and remained within the lower bounds of the norm throughout the treatment period [42]. NK cells are part of the immune surveillance mechanisms for infections and cancer. However, malignancies in CLAD tablet studies have not demonstrated any tumour subtype pattern and the cancer rates were consistent with those found in the general population [43].

Based on the immune function of these cells [44], this suggests that the innate immune system has remained relatively intact [41]. Therefore, the semi-selective nature of CLAD, compared with other highly effective treatments, could limit the incidence of opportunistic infections.

Of the 74 patients studied here, 22.97% developed at least one infection during the follow-up period. In the CLARITY trial [17], except for herpes zoster, the incidence of the most common infections (including severe ones) was similar between the placebo and CLAD-treated groups.

This cohort, compared with patients in the 3.5 mg/kg CLAD arm of the CLARITY study, exhibited a higher EDSS (3.0 ± 1.66 vs. 2.8 ± 1.2) and longer disease duration (9.4 ± 8.8 vs. 7.9 ± 7.2).

The annualised relapse rate (ARR) in this study was 0.68 ± 0.77, while in the CLARITY one, all patients had had at least one relapse in the year before CLAD therapy. Similarly, the MRI disease activity was higher in our cohort with a percentage of Gd+ patients at baseline of 62.3% vs. 31.9% in the CLARITY study [17].

At six months, 9.5% of the patients had a relapse with a statistically significant difference from baseline (*p* < 0.001) and a reduction in ARR from 0.67 to 0.15. At 12 months, 6.7% had a relapse (*p* < 0.001 vs. W0). This reduction in relapse is in line with the CLARITY study [17].

Moreover, at 12 months, 52.9% of the cohort achieved NEDA, which is once again in line with the CLARITY results (54.5%) [17]. No statistically significant correlations were found between LS changes and NEDA outcome. In this regard, by dividing the patients by the previous line of treatment, we found that the Br1 CD19+/CD38+ levels were consistently higher in those who switched to CLAD from first-line treatment vs. second-line treatment (*p* = 0.027). There was, therefore, no subpopulation trend predicting which patients were more inclined to be better responders; however, as in the Comi et al. [40] study, there was no rebound in clinical or MRI disease activity, even after absolute lymphocyte count recovery.

The sNfL levels in the subgroup of 18 patients showed a statistically significant decrease at W24 compared to baseline (*p* = 0.01). These data replicate the results reported in a previous work where 77% of the patients who were treated with subcutaneous CLAD and had high CSF levels of neurofilament light chain at baseline showed normalised values within 12 months [45]. The measurement of sNfL levels represents an important opportunity to verify the effects of this DMT on axonal integrity and evaluate its future potential as an index of treatment outcome. Therefore, studies on the association between longitudinal changes in sNfL and clinical/radiological measures during treatment with DMTs are encouraged.

## 5. Conclusions

To our knowledge, this is the first time that sNfL levels were assessed during treatment with CLAD tablets in a real-life cohort of RMS patients. Despite the limitations of the small sample analysed and the restrictions due to the SARS-COV2 pandemic, which enhanced the difficulty in collecting serial samples, a neuroprotective effect of CLAD can be hypothesised, as suggested by a lowering of sNfL levels, which acts as a biomarker for axonal degeneration; a larger cohort and a longer evaluation period could allow confirmation of this hypothesis.

## Figures and Tables

**Table 1:** Demographic and baseline clinical characteristics of the multiple sclerosis patient cohort.

Characteristic	Entire Cohort (*n* = 74)
Age, mean ± SD (range)	35.2 ± 11.3 (18–56)
Women, *n* (%)	53 (72%)
Disease duration, mean ± SD years (range)	9.4 ± 8.8 (0.12–44.0)
Previous treatment duration, mean ± SD years	10.8 ± 8.9
Previous therapy, *n* (%)	
First line	41 (55%)
Second line	20 (27%)
Naïve	13 (18%)
EDSS, median (IQR)	2.5 (1–7)
ARR (in the previous year), mean ± SD	0.68 ± 0.76
Patients with Gd-enhanced lesions, *n* (%) (in the previous year)	43 (62%)
Patients with new T2 lesions, *n* (%) (in the previous year)	33 (52%)

ARR = annualised relapse rate; EDSS = Expanded Disability Status Scale; IQR = interquartile range; SD = Statistical Deviation.

**Table 2:** No evidence of disease activity (NEDA) results in the multiple sclerosis patient cohort at 12, 18 and 24 months.

Follow-Up	NEDAYes	NEDANo
12 months		
(51 patients) *	*n* = 27	*n* = 24
Previous treatment:		
Naïve, *n* (%)	3 (11%)	3 (12%)
First line, *n* (%)	17 (63%)	11 (46%)
Second line, *n* (%)	7 (26%)	10 (42%)
18 months		
(17 patients) *	*n* = 11	*n* = 6
Previous treatment:		
Naïve, *n* (%)	2 (19%)	1 (17%)
First line, *n* (%)	5 (45%)	5 (83%)
Second line, *n* (%)	4 (36%)	-
24 months		
(8 patients) *	*n* = 5 (62.5)	*n* = 3 (37.5)
First line, *n* (%)	3 (60%)	2 (67%)
Second line, *n* (%)	2 (40%)	1 (33%)

* Number of patients who reached that time of follow-up.

## Data Availability

Not applicable.
